# Adrenalin Chloride in the Treatment of Chronic Nasal, Post Nasal and Pharyngeal Catarrh

**Published:** 1906-11

**Authors:** John C. Warbrick

**Affiliations:** Chicago, Formerly Clinical Assistant to Central Nose, Throat and Ear Hospital, and to Brompton Hospital for Consumption and Diseases of Chest, London, England; Formerly Clinical Assistant to the Nose and Throat Department of Hospital Lariboisiere, Paris, France


For Texas Medical Journal.

### Adrenalin Chloride in the Treatment of Chronic Nasal, Post Nasal and Pharyngeal Catarrh.

BY JOHN C. WARBRICK, M. D., CHICAGO,
Formerly Clinical Assistant to Central Nose, Throat and Ear Hospital, and to Brompton Hospital for Consumption and Diseases of Chest, London, England; Formerly Clinical Assistant to the Nose and Throat Department of Hospital Lariboisiere, Paris, France.

To some extent adrenalin controls the nasal discharge in chronic catarrh, while it allays the congestion of mucous membrane, reduces the swelling of the turbinal tissues, if much hypertrophied, and thus gives a much freer opening through the nostrils and assisting the breathing.

It completely blanches the mucous membrane by contracting the capillaries, thus reducing the local turgescence. In using it as an application in the nostrils, not only the lower part of each cavity should be touched, but the upper part as well, where there may be adhesion of the opposed surfaces, and where a great deal of mucus may collect. The adrenalin will then permit the parts to be seen much better. In doing this, the head should be tilted somewhat backwards to get a more favorable view of the upper part and so the adrenalin chlorid can be applied more easily. In fact the whole of the interior of each nostril can be touched with the solution. The lower part, the upper part, the outer part over the turbinated bodies and the back part as far back as the posterior nares. After this is done a much better view of the parts is to be had, and the indications for treatment much better understood. The strong solution permits of rapid work by acting more quickly on the tissues, but repeated applications of a weaker solution, as 1 in 2000, 1 in 5000, or 1 in 10,000, may serve the purpose just as well.

In some patients the use of strong solution is accompanied by sneezing, coryza, lachrymation or other evidences of slight irritation, but these soon disappear as the adrenalin exerts its characteristic astringent action on the membrane. If there is hypertrophy of the inferior turbinated bodies, the tissues are soon contracted and immediate relief given, especially if the patient has difficulty in breathing through the nose. The action of adrenalin is especially marked by controlling the congestion of the nasal and post-nasal mucous membranes, also the pharyngeal mucous membrane. It renders all operations on the nose and throat bloodless, thus preventing any blood getting into the lungs or stomach. In many cases, where there is a tendency to bleed from the catarrhal conditions present in the nostrils, as ulceration and sometimes necrosis of the tissues and bones, it serves its purpose admirably by checking the bleeding* spot on the septum of left nostril in some of my catarrhal cases not far from the entrance, to which I have applied a plug of cotton saturated with adrenalin and afterwards touched it with strong perchlorid of iron solution. In two cases in particular there has been considerable bleeding from this spot, which I was able to check by first applying a piece of cotton saturated with the perchlorid of iron. In many of my cases the adrenalin has caused a good deal of sneezing which, however, is of favorable omen, as the solution, by contracting the tissues, helps to throw off the catarrhal material in the crypts of the membranes.

In other cases, however, it has produced no effect whatever in the way of sneezing, and I have used both the weaker and the stronger solution. The latter, as I have stated above, is better when more rapid action is wanted, as the blood vessels are constricted sooner and the parts made more accessible to vision while the openings into the nostrils are made freer, such as the Antrum of Highmore, the frontal sinuses and the opening of the nasal duct.

It has been stated in *Merck's Archives*, 1902, that the application of adrenalin chlorid may contract the folds of mucous membrane that normally guard the openings of the cavities which communicate with the nasal chambers, and thus permit the mucus from the nose into these cavities. Patients should, therefore, be warned about blowing their noses except very gently while under the adrenalin treatment, lest muco-purulent matter be forced into the An-

---

*I have noticed a bleeding.

trum of Highmore, or the sphenoidal cells setting up an infectious inflammation there. While the risk of such an accident is small, it still exists, and should be borne in mind. After treating a great many cases of chronic catarrh with excessive discharges and minor discharges, many with hypertrophy of the inferior turbinated bodies, some with atrophy, I have never found it necessary to caution my patients while under treatment, and the majority of them have had occasion to blow their noses a great deal during the course of one sitting alone. In these cases the only warning I have found it necessary to give on account of the excessive blowing of the noses, was the fact that in some subjects of a peculiar diathesis hemorrhage might possibly occur, but from an experience of over one hundred cases I have never had any bleeding occur from this cause or any other trouble from blowing the nose too much. It seems to me, as stated above, that the risk of muco-purulent matter being forced into the Antrum of the sphenoidal cells is very small indeed, especially if the passages are first cleansed by the use of an alkaline solution and cleared of all the accumulated material before using the adrenalin solution. In treating my cases of catarrh I have often applied adrenalin chlorid to the post-nasal space and the throat as well, and have afterwards used a solution of perchlorid of iron with good results.

